# Editorial for “Microbe–Host Interactions in Human Infections”

**DOI:** 10.3390/microorganisms13112548

**Published:** 2025-11-07

**Authors:** Roberta Gaziano, Terenzio Cosio

**Affiliations:** 1 Department of Experimental Medicine, Microbiology and Clinical Microbiology, University of Rome Tor Vergata, 00133 Rome, Italy; 2 Dipartimento di Scienze Biotecnologiche di Base, Cliniche Intensivologiche e Perioperatorie, Università Cattolica del Sacro Cuore, 00168 Rome, Italy

Despite remarkable advances achieved over recent decades, infectious diseases remain a formidable threat to global public health. The escalating prevalence of antimicrobial resistance among pathogens, particularly in the context of emerging and re-emerging zoonotic infections, continues to challenge the medical and scientific communities. Microbial infection represents a highly dynamic and multifaceted interaction between host immune defenses and invading microorganisms [1]. While significant progress has been made in elucidating the complex mechanisms underpinning host–pathogen interactions, our understanding of these processes remains incomplete.

Host–pathogen interactions represent a cornerstone of contemporary microbiology, encompassing the intricate and dynamic interplay of molecular, cellular, and biochemical processes that govern the outcome of infections [1,2]. These interactions, though varying in nature across microbial taxa, share fundamental principles that determine whether a pathogen successfully colonizes the host, elicits immune evasion, or is cleared by innate or adaptive immunity. Host–pathogen interactions are profoundly shaped by the anatomical site of infection, where distinct immune environments, tissue-specific barriers, and microbial communities define the trajectory of disease progression and therapeutic response, making site-specific dynamics a pivotal factor in modern infectious disease management [1,2,3].

In this context, the role of host microenvironments in shaping pathogen behavior has been advanced by studies examining several niches of the human body, such as the composition of airway surface liquid (ASL) in respiratory pathologies. Walsh, Bevan, and Harrison [4] emphasize that the physicochemical properties of ASL—particularly its ion content, mucin concentration, and viscosity—profoundly influence microbial colonization and infection dynamics in the pulmonary tract. Diseases such as cystic fibrosis, chronic obstructive pulmonary disease, and asthma are associated with alterations in ASL that favor pathogen persistence and resistance to mucociliary clearance. This altered milieu not only facilitates the establishment of infections by opportunistic pathogens but also complicates efforts to model respiratory diseases accurately in vitro. The studies included in this Special Issue advocate for a refined understanding of ASL parameters to enhance the fidelity of disease models and to develop more effective interventions tailored to the diseased pulmonary environment. Their findings reinforce the broader notion that pathogen virulence is context-dependent and modulated by host-derived factors, including epithelial surface composition, mucus biochemistry, and local immune activity [4].

Recent investigations into the lung microbiome have further expanded our appreciation of the respiratory tract as a dynamic ecological niche, where both pathogenic microbes and non-pathogenic colonizers significantly influence host immune responses and clinical outcomes. A retrospective study by Hu et al. [5] has expanded the focus beyond pathogenic organisms to examine the prognostic significance of colonizing, non-pathogenic bacteria in pneumonia. In their retrospective study, bronchoalveolar lavage fluid from 483 patients with bacterial, fungal, or viral pneumonia was analyzed using metagenomic next-generation sequencing to assess the distribution of colonizing flora and its impact on prognosis. Distinct colonization patterns were associated with different pneumonia types. Notably, in bacterial pneumonia, *Granulicatella adiacens* was more frequently detected, while *Streptococcus parasanguinis* was less prevalent compared to non-bacterial cases. Fungal pneumonia showed reduced colonization by *Abiotrophia defectiva* but increased levels of *Veillonella parvula*, whereas viral pneumonia was associated with elevated colonization by both *Abiotrophia defectiva* and *Streptococcus mitis.* Among the colonizing species identified, *Rothia mucilaginosa* emerged as particularly beneficial; it was associated with markedly improved clinical outcomes, including shorter hospital stays, decreased ventilator dependence, lower incidence of sepsis, multiple organ dysfunction syndrome (MODS), ICU admission, and reduced mortality. Similarly, *Prevotella melaninogenica* was associated with a reduced risk of MODS and oxygen requirement [5]. These findings challenge the traditional dichotomy between pathogens and harmless commensals, highlighting instead a spectrum of microbial influence shaped by host–pathogen–environmental interactions. The presence of these so-called “beneficial colonizers” appears to modulate cytokine profiles and immune activation levels, suggesting a role for the respiratory microbiota not only in maintaining mucosal homeostasis but also in contributing to resilience against infectious insult. Thus, characterizing and leveraging these microbial players may open new avenues for microbiome-targeted therapeutic interventions in respiratory diseases.

Among the many organisms that exemplify this complexity, especially on the mucosae’s surface, *Candida albicans* stands out as a paradigmatic example of a commensal microbe turned opportunistic pathogen. Under certain conditions, such as immunosuppression or dysbiosis, *C. albicans* can shift from a benign colonizer to an invasive agent capable of inducing a spectrum of diseases, ranging from superficial mucosal infections to life-threatening systemic candidiasis. Its virulence is largely attributed to morphological plasticity—specifically, the transition from yeast to filamentous forms—as well as its ability to form robust biofilms on both biotic and abiotic surfaces. These virulence traits not only facilitate tissue penetration but also confer substantial resistance to antifungal agents and immune effector mechanisms. Moreover, *C. albicans* deploys molecular weapons to evade and/or subvert host defenses and maximize its chance of surviving. Thus, new therapeutic approaches are focusing on pleiotropic molecules that can directly target the fungal pathogen and modulate immune system activation without inducing its hyperactivation.

Recent investigations have sought to exploit our understanding of fungal biology to identify novel therapeutic strategies that circumvent the limitations of current antifungal regimens. One such study evaluated the antifungal efficacy of retinoids—including all-trans retinoic acid (ATRA), trifarotene, and tazarotene—against *C. albicans* [6]. These compounds, which are derivates of vitamin A, have been shown to significantly inhibit fungal proliferation, suppress hyphal morphogenesis, and prevent biofilm development in vitro. Furthermore, in silico molecular docking analyses revealed that retinoids may exert their antifungal effects by targeting key fungal proteins such as heat shock protein (Hsp) 90 and 14α-demethylase, as well as through potential interactions with ergosterol, a critical component of fungal cell membranes [6]. These findings not only upgrade new molecular targets but also suggest a broader role for retinoids as modulators of fungal pathogenicity rather than merely growth inhibitors.

While pharmacological innovation is indispensable, adjunctive biological strategies have also garnered increasing attention. Among them, probiotics have emerged as compelling candidates in the prevention and mitigation of fungal infections, particularly those affecting mucosal surfaces. In this context, *Bacillus coagulans* LMG S-24828, a spore-forming bacterium, has demonstrated notable antagonistic activity against both *C. albicans* and *C. parapsilosis*. Experimental studies employing in vitro co-culture models revealed that both live bacteria and their cell-free supernatant (CFS) significantly curtailed fungal growth, inhibited hyphal development, and reduced adherence to vaginal epithelial cells [7]. The probiotic effect was multifactorial, involving environmental acidification, secretion of antifungal metabolites, co-aggregation with fungal cells, and fortification of epithelial barriers. These findings not only validate the antifungal potential of *B. coagulans* LMG S-24828 but also suggest a feasible prophylactic application, particularly in managing vulvovaginal candidiasis and its recurrent forms [7]. The strain’s ability to survive gastrointestinal transit and colonize mucosal niches further enhances its clinical utility.

Despite the longstanding availability of new treatments and the repurpose of existing molecules, pathogens have evolved to elude immune systems and become resistant to chemotherapies discovered. One emblematic example is *Treponema* (*T.*) *pallidum* sub. *Pallidum*, the etiological agent of syphilis. Syphilis remains a global public health concern, with significant unmet needs in accurate diagnostics, long-term disease monitoring, and effective management of late-stage and recurrent infections. In contrast to the strategies employed by fungal pathogens and their antagonists, bacterial pathogens such as *Treponema* (*T.*) *pallidum* demonstrate a different paradigm of host manipulation, one primarily centered on immune evasion. *T. pallidum* possesses a highly minimalist outer membrane architecture with sparse surface-exposed antigens and lacks traditional virulence factors such as lipopolysaccharide (LPS) or exotoxins [8]. These features render *T. pallidum* elusive to early innate immune recognition. Moreover, the pathogen engages in antigenic variation, particularly of the TprK protein, and induces programmed cell death in T cells via both apoptosis and pyroptosis, thereby weakening the host’s adaptive immune response. Despite this, a robust Th1-mediated immune response does occur during early infection, characterized by the production of interferon-γ (IFN-γ), interleukin-2 (IL-2), and IL-12, which collectively stimulate macrophage activation and facilitate pathogen clearance [8]. Histological analyses have consistently demonstrated the presence of CD4+ and CD8+ T cell infiltrates in syphilitic lesions, with dendritic cells orchestrating antigen presentation and T cell priming. Nevertheless, these responses often fall short of achieving neutering immunity, allowing the spirochete to persist, disseminate, and contribute to the pathogenesis of chronic conditions such as neurosyphilis and cardiovascular syphilis. The expansion of regulatory T-cell populations and the organism’s proclivity for immune-privileged niches further contribute to its long-term survival within the host [8]. The study highlights that the intricate and dynamic interplay between microbes and their human hosts fundamentally shapes the course and outcome of infectious diseases. Advances in dissecting these interactions, from microbial virulence factors and host immune responses to the influence of the microbiome, may open promising avenues for innovative treatments and personalized medicine.

The concept of human anatomical and ecological niches has emerged as a critical determinant in human–host interaction, influencing colonization, persistence, and immune evasion; recognizing and targeting these niche-specific interactions now represents a crucial frontier in the development of precision therapies for infectious diseases [9]. In this intricate context, *Mycobacterium tuberculosis* (Mtb) and HIV coinfection epitomizes a particularly insidious form of host–pathogen interplay, wherein both pathogens collaboratively subvert immune defenses to ensure their survival and proliferation. Mtb exploits macrophage functions by evading lysosomal degradation and establishing persistent intracellular niches, a process that is markedly potentiated during HIV-induced CD4+ T-cell depletion and granuloma disruption [10]. Advances in host-directed therapies (HDTs), particularly those targeting proteolytic pathways, have highlighted promising strategies to enhance immune control. Repurposing saquinavir, an HIV protease inhibitor, has been shown to enhance cathepsin activity within macrophages, thereby restoring antigen presentation and promoting pathogen clearance. Simultaneously, modulation of endogenous protease inhibitors such as cystatin C and F has emerged as a viable approach to reinstate effective microbicidal responses and strengthen adaptive immunity [10]. These novel interventions highlight the therapeutic potential of modulating host proteostasis and immune regulation, particularly in circumventing pathogen-driven immune subversion and drug resistance, thereby offering innovative avenues for integrated management of chronic infectious diseases.

Taken together, these diverse yet interrelated studies underscore the central role of host–pathogen interactions in determining disease outcome and therapeutic efficacy. Whether through direct pharmacologic inhibition of microbial virulence factors, ecological interference by commensal or probiotic organisms, or host-mediated physiological shifts that create permissive environments for infection, it is evident that the pathogenesis of infectious diseases is not a unilateral process. Rather, it results from a continuous and dynamic dialogue between microbial strategies for survival and host mechanisms of defense. Advances in our understanding of these interactions are pivotal not only for the development of targeted therapies but also for informing broader clinical strategies aimed at prevention, early diagnosis, and long-term disease management. Crucially, such insights call for an integrative approach that embraces molecular microbiology, immunology, pharmacology, and systems biology to holistically address the challenge posed by infectious diseases in an era increasingly defined by antimicrobial resistance and emerging pathogens.
